# Profiling Temporal Changes of the Pineal Transcriptomes at Single Cell Level Upon Neonatal HIBD

**DOI:** 10.3389/fcell.2022.794012

**Published:** 2022-03-08

**Authors:** Xin Ding, Tao Pan, Qiuyan Tian, Wenxi Huang, Lauren S Hayashi, Qin Liu, Fuyong Li, Li-Xiao Xu, Po Miao, Xiaofeng Yang, Bin Sun, Chen-Xi Feng, Xing Feng, Mei Li, Jian Huang

**Affiliations:** ^1^ Soochow Key Laboratory of Prevention and Treatment of Child Brain Injury, Children’s Hospital of Soochow University, Suzhou, China; ^2^ Pediatrics Research Institute, Children’s Hospital of Soochow University, Suzhou, China; ^3^ Undergraduate Program, University of Virginia, Charlottesville, VA, United States; ^4^ IRTA Fellow, National Institutes of Health, Bethesda, MD, United States; ^5^ School of Basic Medicine and Biological Sciences, Medical College of Soochow University, Suzhou, China

**Keywords:** hypoxic-ischemic brain damage, pineal gland, single cell RNA sequencing, pinealocyte, astrocyte, microglia, pyroptosis

## Abstract

Neonatal hypoxic-ischemic brain damage (HIBD) often results in various neurological deficits. Among them, a common, yet often neglected, symptom is circadian rhythm disorders. Previous studies revealed that the occurrence of cysts in the pineal gland, an organ known to regulate circadian rhythm, is associated with circadian problems in children with HIBD. However, the underlying mechanisms of pineal dependent dysfunctions post HIBD remain largely elusive. Here, by performing 10x single cell RNA sequencing, we firstly molecularly identified distinct pineal cell types and explored their transcriptome changes at single cell level at 24 and 72 h post neonatal HIBD. Bioinformatic analysis of cell prioritization showed that both subtypes of pinealocytes, the predominant component of the pineal gland, were mostly affected. We then went further to investigate how distinct pineal cell types responded to neonatal HIBD. Within pinealocytes, we revealed a molecularly defined β to α subtype conversion induced by neonatal HIBD. Within astrocytes, we discovered that all three subtypes responded to neonatal HIBD, with differential expression of reactive astrocytes markers. Two subtypes of microglia cells were both activated by HIBD, marked by up-regulation of *Ccl3*. Notably, microglia cells showed substantial reduction at 72 h post HIBD. Further investigation revealed that pyroptosis preferentially occurred in pineal microglia through NLRP3-Caspase-1-GSDMD signaling pathway. Taken together, our results delineated temporal changes of molecular and cellular events occurring in the pineal gland following neonatal HIBD. By revealing pyroptosis in the pineal gland, our study also provided potential therapeutic targets for preventing extravasation of pineal pathology and thus improving circadian rhythm dysfunction in neonates with HIBD.

## Introduction

Neonatal hypoxic-ischemia brain damage (HIBD), which might occur during fetal development, labor or in the postnatal period, often results in permanent deficits such as cerebral palsy and developmental delay (Ferriero, 2004; Shalak and Perlman, 2004). A long-standing observation is that, besides motor and cognitive impairment, neonatal HIDB often results in a much broader spectrum of neurological symptoms (De Vries and Jongmans, 2010).

The sleep-wake cycling (SWC) is established at an early stage after birth in healthy term newborns (Verma et al., 1984; Thornberg and Thiringer, 1990). Classic studies revealed a significant delay of the time of SWC onset by using the amplitude-integrated electroencephalography (aEEG) recording (Osredkar et al., 2005; Takenouchi et al., 2011). Such delayed onset of SWC was even observed in hypothermic-treated neonates with HIBD (Takenouchi et al., 2011). Furthermore, asphyxiated neonates are likely to develop altered sleep organization with decreased active sleep (Scher et al., 2002). Thus, circadian dysfunction and chronic sleep problems are not uncommon features in children with neonatal HIBD. To investigate the underlying mechanisms of HIBD caused circadian problems, we performed whole brain screening and discovered that sleep and circadian rhythm issues in children with HIBD is correlated with the occurrence of cysts in the pineal gland (Ding et al., 2016), an organ known to control circadian rhythm (Borjigin et al., 2012). However, much less is known about the molecular and cellular response of the pineal gland upon neonatal HIBD.

Different from other brain areas, the pineal gland, although located adjacent to the third ventricle of the brain, is mainly composed of pinealocytes rather than neurons. Besides pinealocytes, other cell types including endothelial cells, vascular and leptomeningeal cells (VLMCs), astrocytes, and microglia are discovered in the pineal gland (Møller and Baeres, 2002; Mays et al., 2018). Accumulating evidence showed that astrocytes and microglia cells, both are activated by inflammation, play distinct roles in HIBD pathology during brain development (Liu and McCullough, 2013; Bhalala et al., 2014; Hagberg et al., 2015). An intriguing, yet unresolved, questions is how different cell types contribute to the pineal pathophysiology of neonatal HIBD.

The fact that pineal cysts are formed in children with perinatal HIBD suggests that substantial cell death, largely due to massive inflammation, occurs in the pineal gland. In light of this, a recently identified programmed cell death (PCD), pyroptosis, brought our attention.

Unlike apoptosis, pyroptosis is inflammation associated PCD (Bergsbaken et al., 2009). Mechanistically, in the canonical model, inflammatory ligands trigger the formation of inflammasomes, which will then recruit and activate caspase-1. Activated caspase-1 cleaves and activates gasdermin D (GSDMD) to form pores of the cell membrane that leads to cell death. Recent studies showed that pyroptosis is actively engaged in the pathology of the traumatic brain injury (TBI), neurodegenerative diseases, and infections of the central nervous system (De Nardo et al., 2014; Broderick et al., 2015; Guo et al., 2015). Notably, microglial pyroptosis is observed in the cerebral cortex upon neonatal HIBD (Lv et al., 2020; Tan et al., 2021). However, whether and how microglia activated pyroptosis engages in the pineal pathology upon neonatal HIBD remains unknown.

In this study, we sought out to profile transcriptome changes at single cell level in the pineal gland at two time points after neonatal HIBD. By examining transcriptome changes of distinct cell types, this study aims to explore a dynamic and formative image of molecular and cellular events that occur along the time axis post neonatal HIBD. Furthermore, we revealed microglia pyroptosis as an important component of pineal pathophysiology upon neonatal HIBD. Our study thus provided novel insights into the molecular and cellular pineal pathophysiology of neonatal hypoxic injury. This will further benefit the identification of potential therapeutic targets to treat circadian dysfunction observed in children with neonatal HIBD.

## Materials and Methods

### Establishment of Neonatal Hypoxic-Ischemic Brain Damage Model

In the whole study, all animal surgeries were approved by the ethical committee of Soochow University and performed in accordance with the institution guidelines for animal use and care. Pregnant pathogen free (SPF) Sprague Dawley (SD) rats were obtained from JOINN New drug research center co. LTD. (Suzhou, China). After birth, neonatal rats were freely fed by their mums in the animal room (12-12 h dark-light cycle with constant room temperature maintained at 25 ± 2°C). Rats with mixed gender were randomly assigned into sham or HIBD group. We used 15–20 rats (average weight: 15–20 g) per time point in both sham and HIBD groups. The establishment of neonatal HIBD model was modified from Rice-Vannucci method (Rice et al., 1981). In brief, at neonatal day 7, under anesthesia (1.5% isoflurane), we made a double-layer ligation (No. 3–0 silk thread) of the left common carotid artery under a surgical microscope. After surgery, we immediately transferred neonatal pups to a low-oxygen chamber (a gas mixture of moist 8% nitrogen-oxygen, 1.5 L/min, 37°C). For sham control, we exposed the left common carotid artery under isoflurane without making any ligation. Pups were then placed in a 37°C warm pad. Two hours later, we returned animals receiving sham or HIBD surgeries to their mums. To exclude effects of circadian changes of gene expression, we performed all sham or HIBD surgeries between 4:00–6:00 p.m. and obtained pineal tissues strictly at 24 and 72 h post surgery. To validate brain damages caused by this model, we euthanized animals (CO_2_) receiving sham or HIBD surgeries at 24 and 72 h post injury, prepared transverse sections (1 mm thickness) of the brain or the pineal gland and stained with 4% 2,3,5-trphenyltetrazolium chloride (TTC, sigma), which reveals infarcted areas (Supplementary Figure S1).

### Preparation of Pineal Gland Single Cell Isolation

At 24 h (P8) and 72 h (P10) post surgeries, neonatal rats were euthanized, and the pineal glands were immediately dissected out from their brains. The protocol of generating pineal single cell solution was adapted from previous publications (Schaad et al., 1993; Mays et al., 2018). In brief, we first made and preheated (5% CO_2_ at 37°C, 0.5 h) the digestion solution [20 U/ml papain (Sigma, 9001-73-4), dnase 100 mg/L (Sigma, DN-25), 0.2 U/ul SuperaseIn RNase Inhibitor (Thermofisher Scientific, AM2694) in 1x HBSS]. For each time point/condition, pineal glands from multiple animals were pooled and added with preheated papain solution, incubated at 37°C. During this period, we applied intermittent agitation in each 10 min by performing gentle titration using a 1 ml pipette tip. After 45 min, the digestion solution containing pineal cells were filtered by a pre-wetted 70 μm strainer (FisherScientific, 08-771-2) and centrifuged (300 g, 4 C) for 5 min. Pellets were then resuspended in 1xPBS (0.1% BSA) for single cell sequencing.

### Single-Cell Library Preparation and RNA-Sequencing

Isolated pineal gland cells at different time points were partitioned into nanoliter-scale Gel Bead-In-Emulsions (GEMs) (10X Genomics; Pleasanton, CA). We used Chromium Single Cell 30 Reagent Kits v2 to generate full-length, barcoded cDNAs for PCR amplification. The final libraries contain standard Illumina paired-end constructs which begin and end with P5 and P7. Sample index sequences are incorporated as the i7 index read. Read 1 and Read 2 are standard Illumina^®^ sequencing primer sites used in paired-end sequencing.

### Single-Cell Data Generation, Pre-processing, Identification of Cell Clusters

change the original sentence to “The data presented in the study are deposited in the Bioproject (NCBI) repository, accession number PRJNA743566”. We used the Cell ranger (http://support.10xgenomics.com/single-cell/software/overview/welcome) to align the exonic reads to the genome and perform quality control. After that, we used the Seurat package (Hao et al., 2021) to perform further filtering (Feature RNA: 250-6000, mitochondria: <0.2). Next, we used Uniform Manifold Approximation and Projection (UMAP) to visualize cell clusters. Two cell types: blood cells (Hba-a1^+^ & Hba-a2^+^) and oligodendrocytes (MBP^+^ & Plpr^+^) that form myelin sheet of innervating axons were excluded for further analysis. After filtering, 32,425 (24 h sham), 16,379 (24 h HIBD), 8491(72 h sham), and 16,916 (72 h HIBD) pineal gland cells were included for next level analysis. Heat-maps, violin plots and volcano plots to identify relative gene expression levels in distinct cell clusters at different time points post neonatal HIBD.

The analysis of cell type prioritization using Augur is completed by strictly following a step-by-step protocol (Squair et al., 2021).

### RNA Quantification and Western Blotting

To validate results of the single cell sequencing, we euthanized (CO_2_) animals receiving sham or HIBD at 24 and 72 h post injury and immediately dissected out the pineal glands (n = 5 for each time point). Next, we extracted total RNAs (Trizol, Invitrogen) from homogenized tissues. To quantify relative RNA levels, total pineal RNAs were reversely transcribed (Superscript III, Invitrogen) and amplified by quantitative PCR (CFX Connect, BioRad, Sybr Green master mix, Thermofisher, United States). We used *Gapdh* as a loading control. The primers used were: *Aanat* forward: 5′- CATCCCTTCCTGGCTCC; *Aanat* reverse: 5′- GGGAACTAGGGAGGCAG (length: 153 bp); *Asmt* forward: 5′- GAC​GTT​GGA​ATC​AGA​GGT​CAG; *Asmt* reverse: 5′- CTT​CCA​GTC​TCC​TTG​CTT​GAG (length: 100 bp); *Ccl3* forward: 5′- GAA​GTC​TTC​TCA​GCG​CCA​TA; *Ccl3* reverse: 5′- AAA​GGC​TGC​TGG​TCT​CAA​A (length: 112 bp); *Ccl4* forward: 5′- CTA​TGA​GAC​CAG​CAG​CCT​TT; *Ccl4* reverse: 5′- CAA​CTC​CAA​GTC​ATT​CAC​ATA​CTC (length: 121 bp); *Gapdh* forward: 5′-GAC​ATG​CCG​CCT​GGA​GAA​AC; *Gapdh* reverse: 5′-AGC​CCA​GGA​TGC​CCT​TTA​GT (length: 193 bp).

To quantify protein levels, western blotting was performed as previously described (Yang et al., 2017). In brief, pineal glands from mice receiving sham or HIBD surgeries were collected and lysed in 500 μl of homogenization buffer. The primary antibodies used were: 1) rabbit polyclonal anti-NLRP-3 (Abcam, ab91413, 1:500), 2) rabbit polyclonal anti-caspase-1 (Abcam, ab 1872, 1:500), 3) rabbit polyclonal anti-GSDMD (Abclonal, A10164, 1:400), 4) rabbit monoclonal anti-cleaved N-terminal GSDMD (Abcam, ab215203, 1:1,000) and 5) mouse monoclonal β-actin (Sigma, A1978, 1:200). The secondary antibody used were: horseradish peroxidase-conjugated rabbit/mouse anti-rat IgG. We developed the membrane by chemiluminescence kit (SuperSignal West Pico; Pierce) and imaged on a ChemiDoc XRS System (Biorad, Hercules, CA). Band intensities were quantified using Quantity One software (Biorad).

### Immunohistochemistry and TUNEL

We euthanized (CO_2_) animals receiving sham or HIBD surgeries at 24 and 72 h post injury, performed cardiac perfusion (4% paraformaldehyde), and prepared transverse cryosections of the pineal gland at the thickness of 30 µm. For immunohistochemistry, we incubated sections with a rabbit polyclonal antibody against Ki67 (Abcam, ab15580, 1:200), a mouse monoclonal antibody against vimentin (Abcam, ab8978, 1:500), a rabbit polyclonal antibody against Iba1 (ThermoFisher Scientific, 10904-1-AP, 1:500), or a mouse monoclonal antibody against caspase-1 (14F468) (Santa Cruz, sc-56036, 1:400) at 4°C overnight. After 3 times of wash in 1x PBS (with 0.5% TrintonX-100), sections were incubated with Alexa Fluor 488-conjugated goat anti rabbit or mouse secondary antibodies (Invitrogen) for 2 h at room temperature. In all cases, the antibody specificity was validated in previous publications (Fu et al., 2015; Choi et al., 2019; Kon et al., 2021; Xing et al., 2021). In addition, we performed immunostaining without primary antibody incubation and detected no fluorescent signals. TUNEL assay was performed according to the manual (TUNEL-FITC, Abcam, ab66108). DNase I treated or PBS treated sildes were used as a positive and negative control of TUNEL staining, respectively. Transverse sections were imaged using a confocal laser-scanning microscope (Zeiss 710). We used ImageJ (NIH) to quantify the relative fluorescent intensity of vimentin or caspase-1 by calculating the mean fluorescent intensity of pineal sections (background intensity subtracted) in arbitrary units. To quantify Ki67^+^, TUNEL^+^ or Iba1^+^/Caspase-1^+^ cells, we counted those (co-labeled with DAPI) five transverse sections crossing the whole pineal gland for individual animal. In both cases, three or five animals were used from sham or HIBD groups. All quantification was performed blindly.

### Statistical Analysis

In all figure panels, data were presented as mean ± SEM, with original data points plotted. We applied student’s t test, One-way ANOVA followed by Bonferroni’s correction for two or multiple group comparisons. For all statistics, **, *p* < 0.01, *, *p* < 0.05, no statistical significance as n. s. (no significance).

## Results

### scRNA-Seq Profiling of Pineal Gland at Different Time Points Post Neonatal HIBD

**FIGURE 1:**
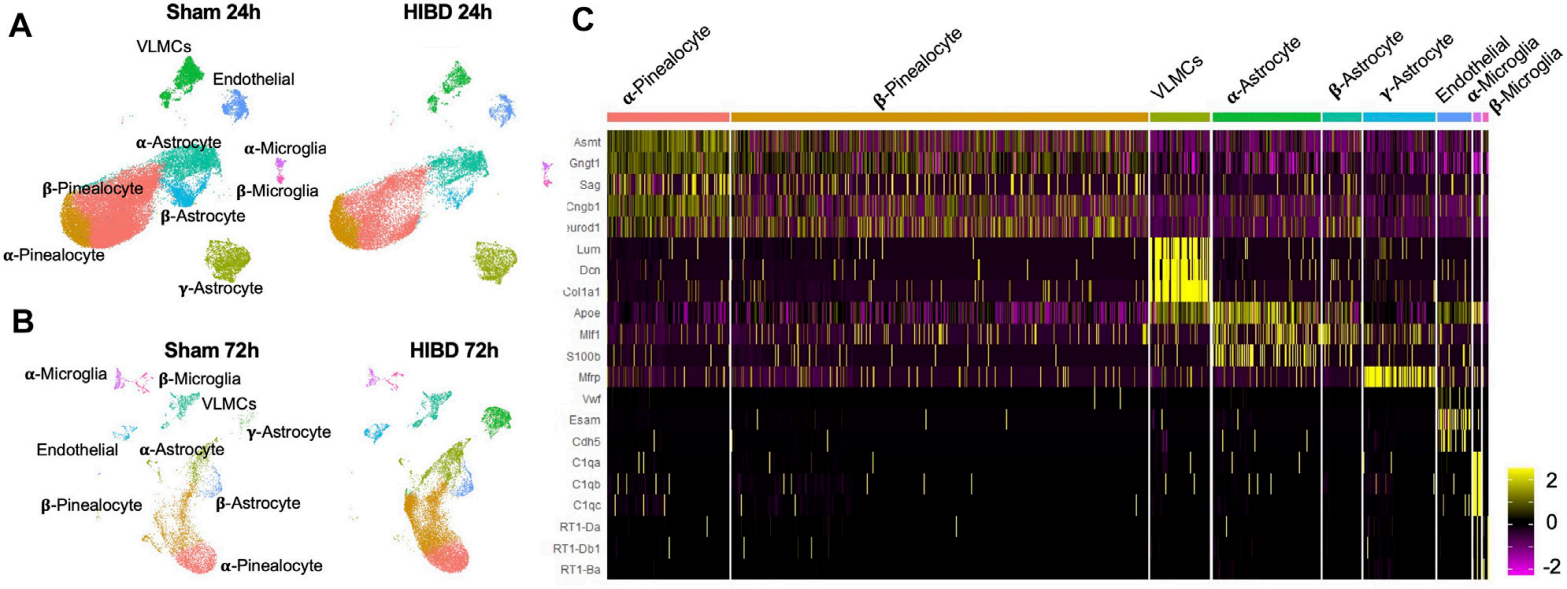
Transcriptomic characterization of pineal gland at single cell level post neonatal HIBD **(A,B)** Uniform Manifold Approximation and Projection (UMAP) visualization of rat pineal gland cells collected at 24 and 72 h post sham or neonatal HIBD **(C)** Heatmap of expression values of characteristic genes for distinct cell types.

To identify temporal changes of transcriptomes of different cell types, pineal glands from neonatal rats receiving sham or HIBD surgeries were subject to 10x-single cell sequencing at 24 and 72 h post injury, respectively. These two time points were chosen to represent different clinical stages of perinatal HIBD (Sarnat and Sarnat, 1976; Allen and Brandon, 2011). Because during the first 2 weeks after birth, the pineal gland is still undergoing proliferation and differentiation (Steinberg et al., 1981; Calvo and Boya, 1983; Calvo et al., 2004), we performed the Uniform Manifold Approximation and Projection (UMAP) analysis separately for individual time points (Figure 1A). Consistent with previous studies (Mays et al., 2018), our data revealed 5 cell types [pinealocytes, vascular and leptomeningeal cells (VLMCs), astrocytes, endothelial cells, and microglia] present in the pineal gland both under sham or HIBD conditions (Figures 1A,B). Dependent on the differential expression of distinct marker genes, the pinealocytes, astrocytes and microglia cells are further divided into multiple subtypes (Figures 1A–C). We will elaborate transcriptome changes of distinct cell subtypes in the following sections.

### Effects of Neonatal HIBD on Cellular Components in the Pineal Gland

**FIGURE 2:**
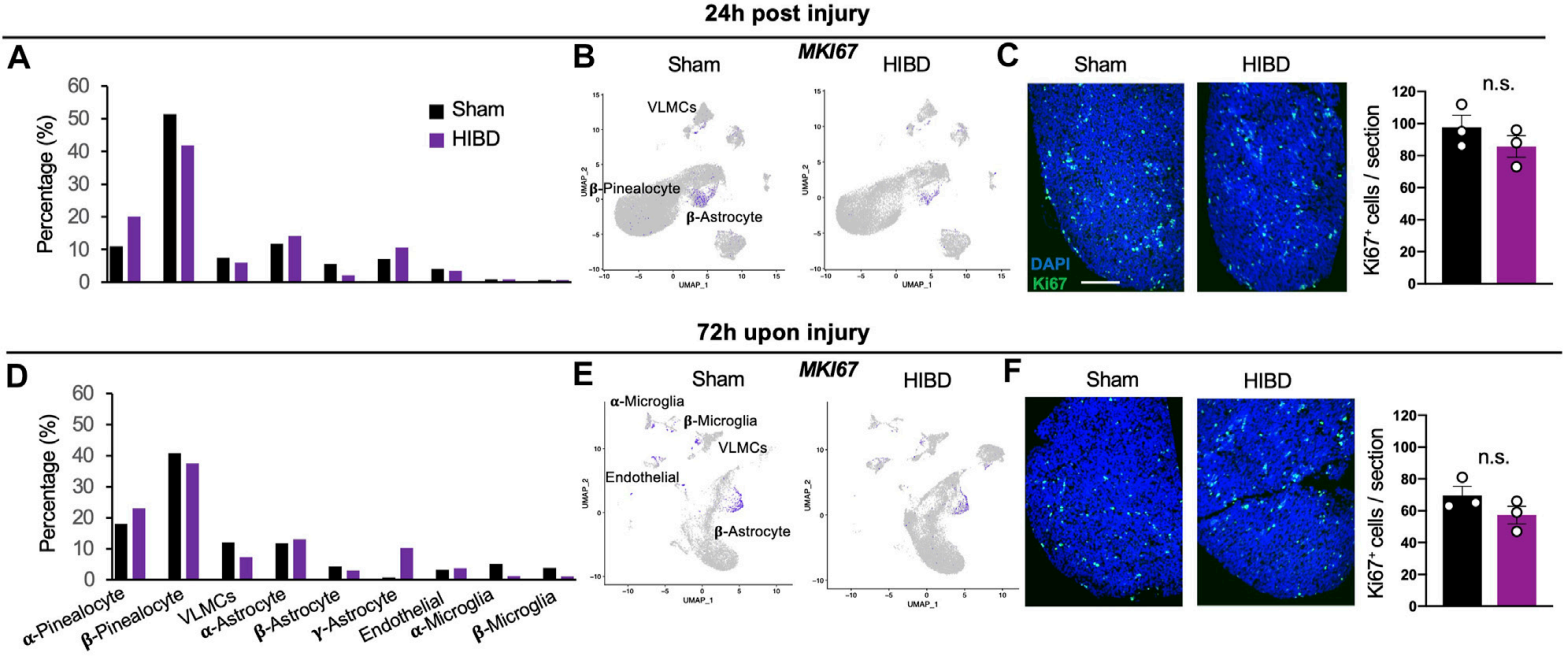
Cell composition and proliferation of the pineal gland post neonatal HIBD **(A,D)** Relative composition of distinct cell types in total pineal cells (%) at 24 h **(A)** or 72 h **(D)** post sham or neonatal HIBD, respectively **(B,E)** Feature plots of Ki67 among distinct cell types of the pineal gland at 24 h **(B)** or 72 h **(E)** post sham or neonatal HIBD, respectively **(C,F)** Representative images of transverse sections of the pineal gland with immunostaining against Ki67 and quantification at 24 h **(C)** or 72 h **(F)** post sham or neonatal HIBD, respectively. Scale bar: 200 μm n = 3,3 for sham and HIBD animals, five sections crossing the pineal gland were used for individual animals, Student’s *t* test, n. s, no statistical significance.

When comparing the cellular components of the pineal gland in animals receiving sham injuries, we discovered an increase of molecularly defined α-pinealocytes and microglia, and a decline of non α-astrocytes between the 24 h (corresponding to P8) and 72 h (corresponding to P10) groups (Figures 2A,D). Such cell type dynamics were in consistent with our own observation during postnatal pineal development (unpublished).

Next, we went further to investigate whether and how neonatal HIBD affects the cell type composition of the pineal gland (Figures 2A,D). While the proportion of certain cell types (e.g. endothelial cells) remained unchanged, that of multiple cell types and subtypes were drastically affected. First, the total fraction (about 65%) of pinealocytes among all cell types was relatively unaffected by HIBD (Figures 2A,D). On the basis of molecular identification of α- and β-pinealocytes (higher expression of *Asmt*), the abundance of α-relative to β-pinealocytes was expanded in animals receiving HIBD (Figures 2A,D). Second, α- or β-subtypes underwent moderate changes at both time points, respectively (Figures 2A,D). In contrast, a drastic expansion of γ-astrocytes was observed at 72 h post HIBD (Figure 2D). γ-astrocytes is marked by Membrane frizzled-related protein (MFRP) gene, a spatially specific marker for astrocytes in the caudal brain (Cuevas-Diaz Duran et al., 2019) (Figure 1C). Third, while the fraction of two microglia subtypes was not affected at 24 h post HIBD, that of both showed substantial reduction at 72 h post HIBD (Figures 2A,D).

Previous studies showed that the pineal gland undergoes postnatal cell proliferation within the first 2 weeks after birth (Calvo et al., 2000; Calvo et al., 2004). We then investigated whether neonatal HIBD affected proliferation by assessing the expression of Ki67, a well-established marker of cell proliferation (Gerdes et al., 1984). The most prominent proliferation, although showing no overt differences between sham and HIBD groups at both 24 and 72 h post injury, was seen in β-astrocytes (Figures 2B,E). To validate these data, we stained transverse sections of the pineal gland from animals receiving sham or HIBD surgeries using an antibody against Ki67. The immunostaining revealed moderate cell proliferation at 24 and 72 h post injury (Figures 2C,F). Consistent with results from the single cell sequencing, such cell proliferation showed no significant difference between the sham and HIBD conditions at both time points post injury (Figures 2C,F).

### Cell Type Prioritization in the Pineal Gland Upon Neonatal HIBD

**FIGURE 3:**
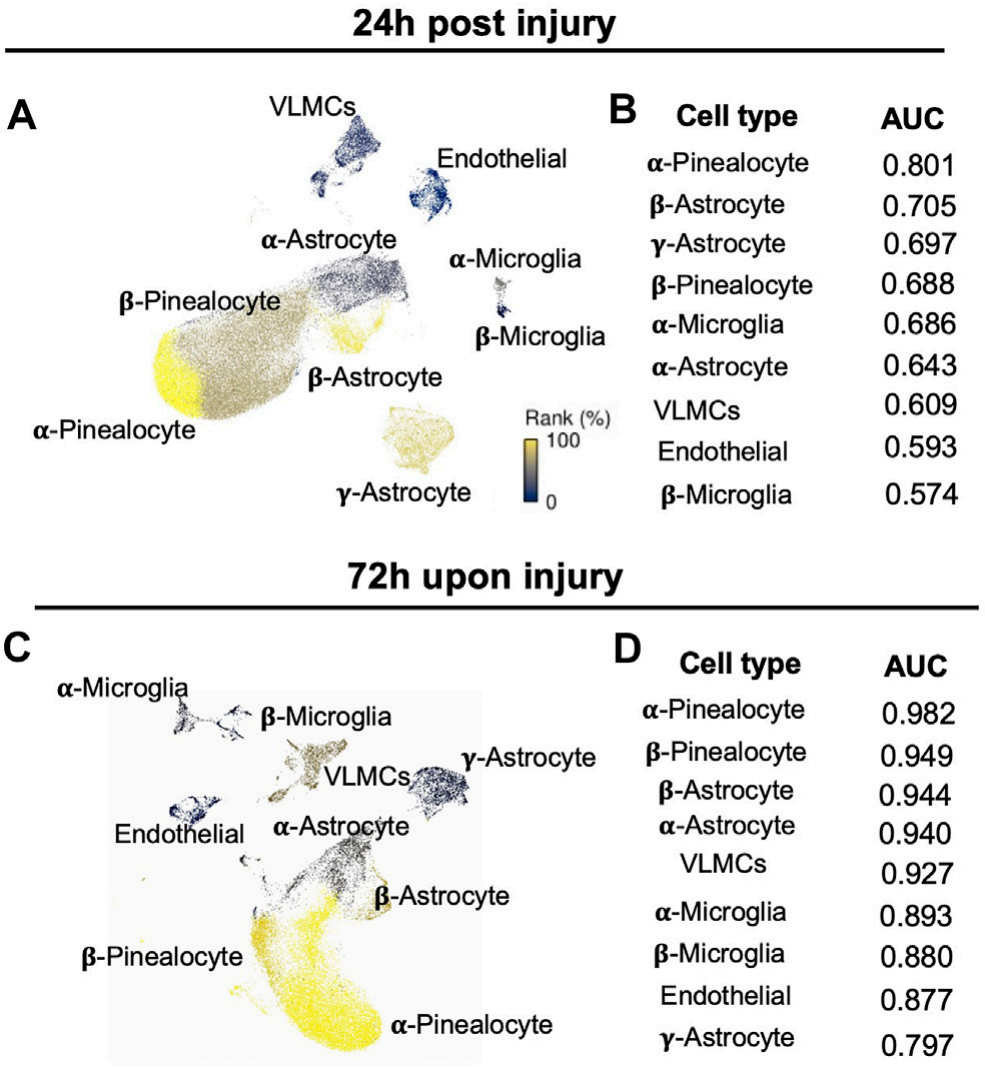
Identification of cell type prioritization in the pineal gland upon neonatal HIBD **(A,C)** UMAP visualization of distinct cell types colored by Augur cell type prioritization at 24 h **(A)** or 72 h **(C)** post sham or neonatal HIBD, respectively **(B,D)** Values of the area under the receiver operating characteristic curve (AUC) of distinct cell types colored by Augur cell type prioritization at 24 h **(B)** or 72 h **(D)** post sham or neonatal HIBD, respectively.

Given that the response to the pathological perturbation is highly cell-type-specific, we went further to investigate which cell types are most responsive in the context of neonatal HIBD. We applied a novel algorithm (Augur) for cell type prioritization (Skinnider et al., 2021). This method defines the prioritization on the basis of molecular response of each cell types rather than relative number of differentially expressed genes. Our results showed that overall response among different cell types was more dramatic at 72 vs 24 h (Figures 3A–D). Within different cell subtypes, α-pinealocytes, along with β-pinealocytes were mostly affected at both time points (Figures 3A–D). Next to pinealocytes, β-astrocytes were calculated as mostly affected astrocytes upon injury (Figures 3A–D), probably due to its proliferative characteristics (Figures 2B,D).

### Transcriptome Changes of α and β-pinealocytes at Different Time Points Post Neonatal HIBD

**FIGURE 4:**
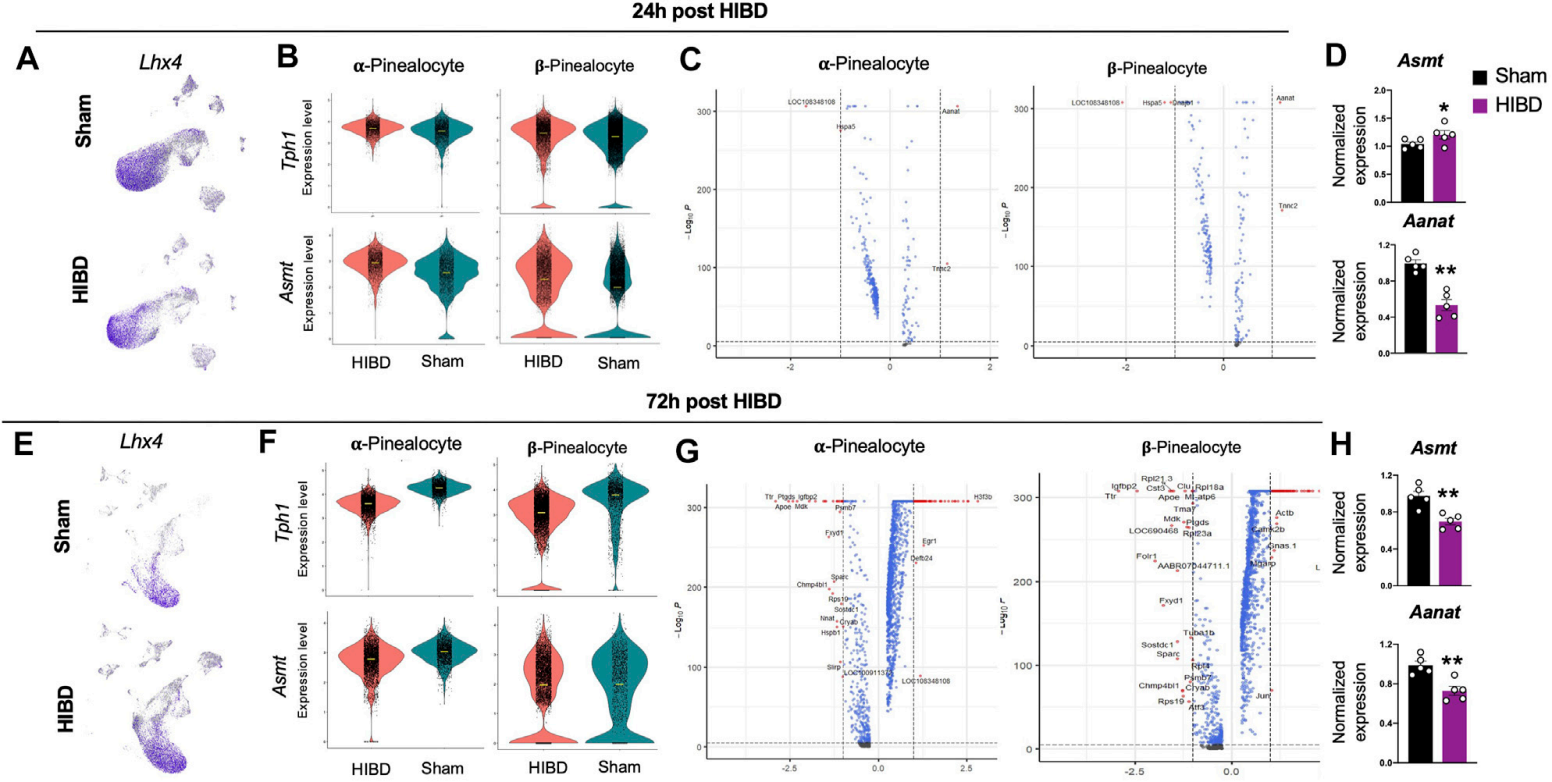
Transcriptome changes in pinealocyte subtypes post neonatal HIBD **(A,E)** Feature plots of a pinealocyte specific transcriptional factor (*Lhx4*) over total pineal populations at 24 h **(A)** or 72 h **(E)** post sham or neonatal HIBD, respectively **(B,F)** Violin plots showing expression differences of characteristic genes (*Tph1* and *Asmt*) at 24 h **(B)** or 72 h **(F)** post sham or neonatal HIBD, respectively **(C,G)** Volcano plots highlighting genes with significant expressional changes between sham and HIBD in both subtypes at 24 h **(C)** or 72 h **(G)** post sham or neonatal HIBD, respectively. Red dots: < adjusted *p* value (0.0001) and >2 fold; Blue dots: < adjusted *p* value (0.0001) with <2 fold; Gray dots: no significance **(D,H)** Quantification of pineal RNA expression of *Asmt* and *Aanat* at 24 h **(D)** or 72 h **(H)** post sham or neonatal HIBD, respectively. n = 5,5 for sham and HIBD animals, fold changes of RNA expression were first normalized to those of *GAPDH*, and then to those in the sham. Student’s t test, ** or *, *p* < 0.01 or 0.05.

Since pinealocytes were mostly affected (Figure 3) post neonatal HIBD, we next sought out to examine how they were affected by neonatal HIBD at single cell level. Marked by a transcriptional factor (*Lhx4*) (Rath et al., 2009; Hertz et al., 2020), pinealocytes are a predominant component of the pineal gland (Figures 4A,E). Consistent with previous findings (Pevet, 1977; Calvo and Boya, 1984; Mays et al., 2018), we separated pinealocytes into α- and β-subtypes, based on their differential expression of *Acetylserotonin O-Methyltransferase (Asmt)*, a rate-limiting enzyme of melatonin synthesis (Figure 1C).

We first examined the expressional changes of two mRNAs: *Tryptophan Hydroxylase 1(Tph1)* and *Asmt*, whose protein products catalyze the first and the last step of melatonin synthesis. We observed a transient up-regulation of *Tph1* and *Asmt* in both α- and β-pinealocytes at 24 h post neonatal HIBD (Figures 4B,F). We then explored global RNA changes in distinct pinealocyte subgroups. Consistent with results from cell type prioritization analysis (Figure 3), transcriptomes of both α- and β-pinealocytes underwent more dramatic changes at 72 h when compared to those at 24 h (Figures 4C,G).

Next, we quantified expression changes of candidates that are key factors controlling the rhythmic synthesis of melatonin using quantitative RT-PCR (Roseboom et al., 1996; Klein, 2007; Ho and Chik, 2010). The expression of *Asmt* showed a transient increment at 24 h post injury, but was significantly reduced at 72 h post injury (Figures 4D,H). The expression of *Aralkylamine N-Acetyltransferase (Aanat)* exhibited significant reduction at both time points post injury (Figures 4D,H). These results suggested that the synthesis and release of melatonin were significantly compromised post neonatal HIBD. In consistent with this, our previous findings revealed profound impairment of Aanat and melatonin levels in the pineal gland post neonatal HIBD (Yang et al., 2017).

### Transcriptome Changes of Distinct Astrocyte Subtypes at Different Time Points Post Neonatal HIBD

**FIGURE 5:**
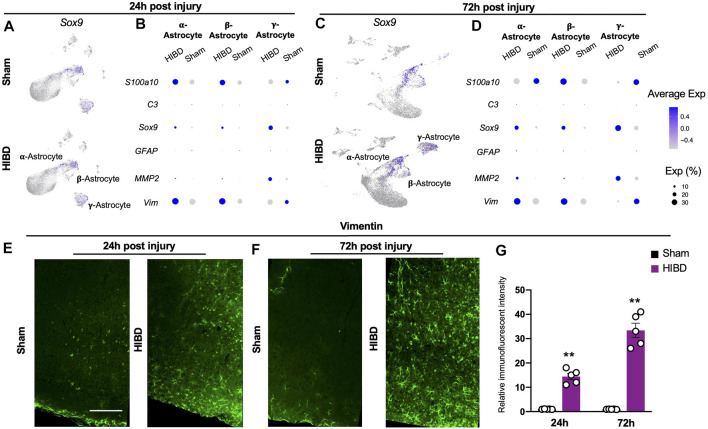
Expression changes of characteristic genes in distinct astrocyte subtypes post neonatal HIBD **(A,C)** Feature plots of an astrocyte specific transcriptional factor (*Sox9*) over total pineal populations at 24 h **(A)** or 72 h **(C)** post sham or neonatal HIBD, respectively **(B,D**) Bubble plots showing expression levels of reactive astrocyte related genes at 24 h **(B)** or 72 h **(D)** post sham or neonatal HIBD, respectively. Note, the size of dots represents the abundance of cells expressing of a given gene; the color scale represents normalized expression level for a given gene across all cells **(E–G)** Representative images of transverse sections of the pineal gland with immunostaining against Vimentin and quantification **(G)** at 24 h **(E)** or 72 h **(F)** post sham or neonatal HIBD, respectively. Scale bar: 200 μm n = 5,5 for sham and HIBD animals, five sections crossing the pineal gland were used for individual animals, Student’s *t* test, **, *p* < 0.01.

Consistent with previous findings, we divided astrocytes, marked by transcriptional factor *Sox9* (Ge et al., 2021) (Figures 5A,C), into three subtypes, on the basis of the differential expression of S100b, ApoE, and MFRP (Zang et al., 1985; Suzuki and Kachi, 1995; Mays et al., 2018) (Figure 1C).

Next, we investigated whether and how these 3 distinct astrocyte subtypes responded to neonatal HIBD. While all three astrocyte subtypes increased the expression of *Sox9*, they exhibited differential expression of reactive astrocytes markers (Figures 5B,D). In detail, *Vimentin and S100a10* were up-regulated in α- and β-astrocytes at 24 and 72 h post injury (Figures 5B,D).

In contrast, *Matrix metalloproteinase*-2 (*MMP2*), which promotes wound healing (Hsu et al., 2006), was preferentially up-regulated in γ-astrocytes (Figures 5B,D). Notably, the *Glial fibrillary acidic protein (GFAP)* and the *complement factor 3 (C3)*, two commonly identified reactive astrocyte markers (Escartin et al., 2021), were not revealed by single cell sequencing (Figures 5B,D). Using immunostaining, we validated that Vimentin showed progressive up-regulation in the pineal gland over the time course post HIBD (Figures 5E–G). Taken together, these results revealed pattern specific activation of astrocytes post ischemia injury.

### Transcriptome Changes of Distinct Microglia Cells at Different Time Points Post Neonatal HIBD

Besides astrocytes, microglia cells as another component of glia cells, play a crucial role during pineal development (Ibañez Rodriguez et al., 2016). Consistent with a previous report (Mays et al., 2018), microglia cells, marked by a transcriptional factor *lymphoblastic leukemia associated hematopoiesis regulator 1 (Lyl1)*, were divided into two subgroups, on the basis of their differential expression of complements (e.g. C1qa, C1qb, C1qc) (Figure 1C).

**FIGURE 6:**
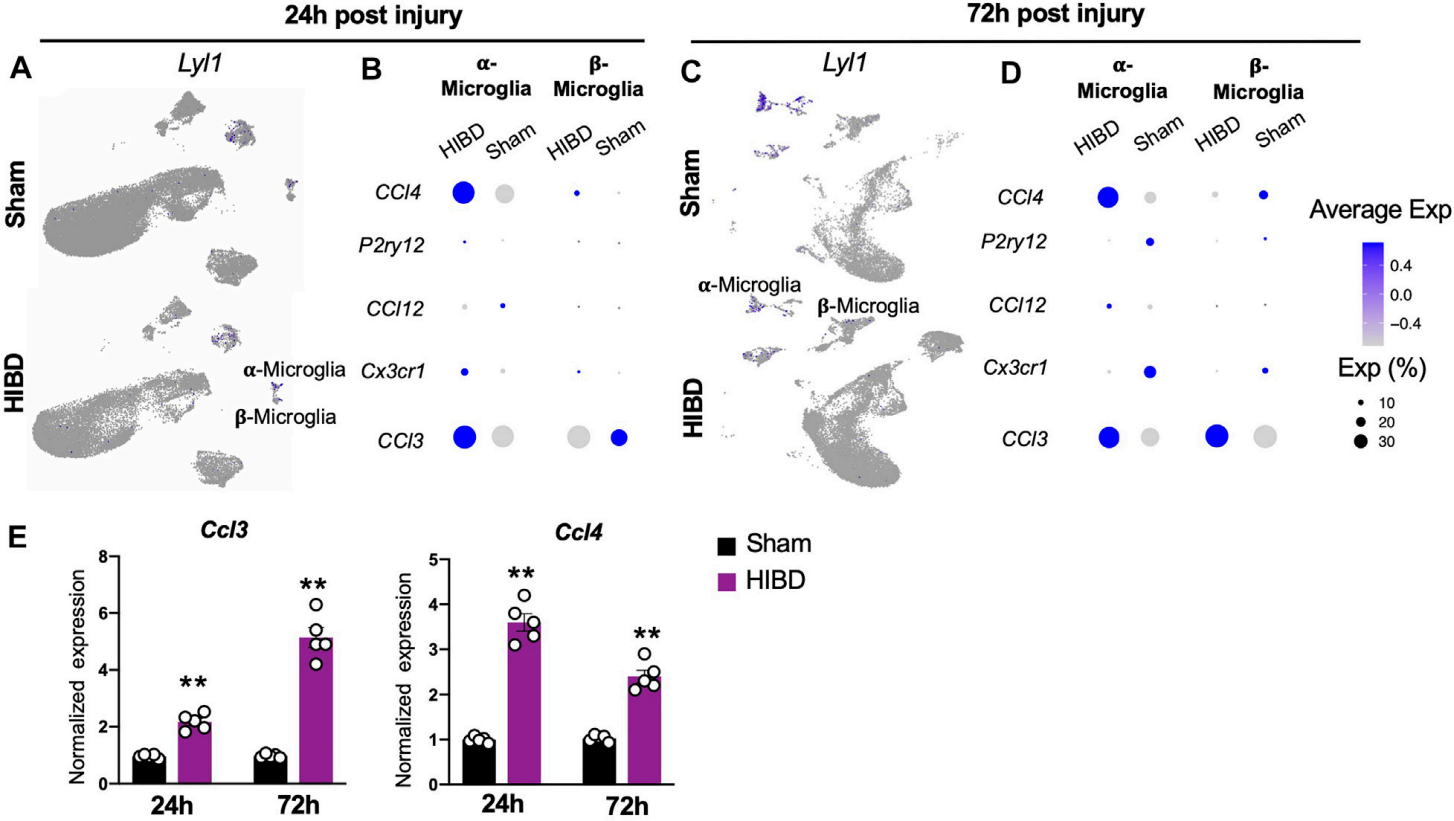
Expression changes of characteristic genes in distinct microglia subtypes post neonatal HIBD **(A,C)** Feature plots of a microglia specific transcriptional factor (*Lyl1*) over total pineal populations at 24 h **(A)** or 72 h **(C)** post sham or neonatal HIBD, respectively **(B,D)** Bubble plots showing expression levels of inflammation related genes at 24 h **(B)** or 72 h **(D)** post sham or neonatal HIBD, respectively **(E)** Quantification of pineal RNA expression of *Ccl3* and *Ccl4* at 24 h or 72 h post sham or neonatal HIBD, respectively. n = 5,5 for sham and HIBD animals, fold changes of RNA expression were first normalized to those of *GAPDH*, and then to those in the sham. Student’s *t* test, **, *p* < 0.01.

Extensive studies have shown that microglia cells actively respond to neonatal brain with HIE (Zhu et al., 2005; Ferrazzano et al., 2013; Lv et al., 2020). We therefore investigated how two molecularly distinct pineal microglia subtypes engage in the neonatal HIBD pathology. Consistent with previous findings (Salter and Stevens, 2017; Hammond et al., 2019), we observed a transient up-regulation (at 24 h post HIBD), followed up with declined expression (at 72 h post HIBD) of the canonical microglial markers *P2ry12* and *Cx3cr1* (Figures 6B,D) in both subtypes. Next, we went further to examine the expression of inflammation signals that are known to be associated with injury-induced-microglia activation (Hammond et al., 2019). Notably, we observed up-regulation of both *Chemokine (C-C motif) ligand 4 and 3* (*Ccl4* & *Ccl3*) in α-microglia cells at 24 and 72 h post HIBD (Figures 6B,D). In contrast, β-microglia cells showed a delayed activation of *Ccl3*, but not *Ccl4* (Figures 6B,D). Using quantitative RT-PCR, we validated the up-regulation of both *Ccl3* and *Ccl4,* two microglia specific cytokines, in the pineal gland at both 24 and 72 h post HIBD (Figure 6E). These results showed that both subtypes of pineal microglia responded to neonatal HIBD, with activation of specific transcriptional programs.

### Microglial Pyroptosis in the Pineal Gland at Different Time Points Post Neonatal HIBD

**FIGURE 7:**
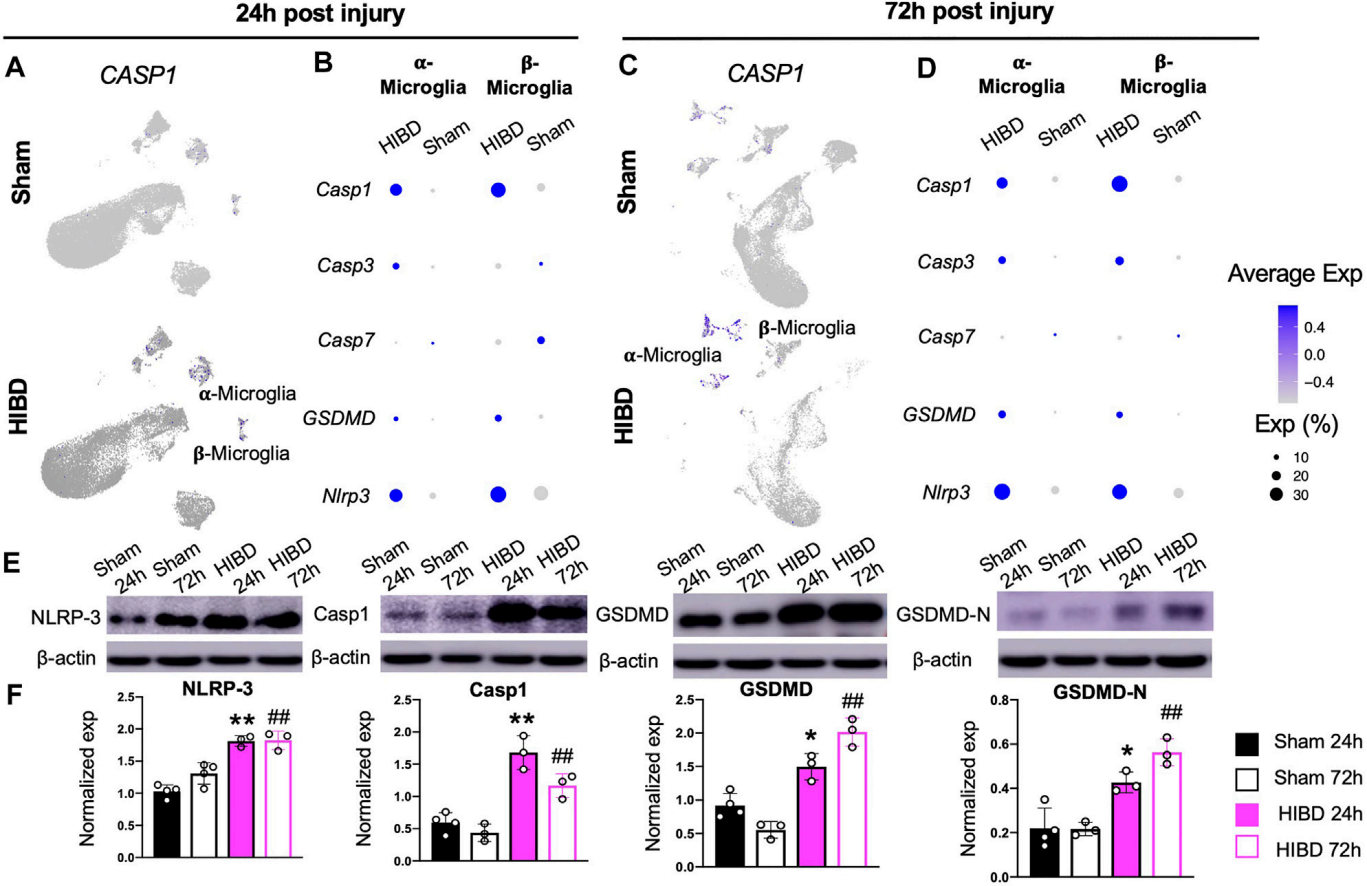
Microglial pyroptosis post neonatal HIBD **(A,C)** Feature plots of a pyroptosis specific factor (*Caspase-1*) over total pineal populations at 24 h **(A)** or 72 h **(C)** post sham or neonatal HIBD, respectively **(B,D)** Bubble plots showing expression levels of pyroptosis and apoptosis related genes at 24 h **(B)** or 72 h **(D)** post sham or neonatal HIBD, respectively **(E–F)** Western blotting **(E)** with quantification **(F)** of NLRP-3, Capase-1, GSDMD, and GSDMD-N. in F, for each lane, the expression of a given protein is first normalized to that of β-actin. ** or *, *p* < 0.01 or 0.05 (sham and HIBD 24 h), ## (sham and HIBD 72 h), *p* < 0.01, n = 3 for each condition, one-way-ANOVA with Bonferroni’s multiple-comparisons *post hoc* test.

When we examined changes of cellular components of the pineal gland post neonatal HIBD, a prominent change is the sharp decrease of both α- and β-microglia cells (Figure 2C), which actively respond to neonatal HIBD (Figure 6). These results suggested inflammation induced programmed cell death (PCD) might occur within microglia populations. To test this, we first examined the expression changes of key molecules involved in PCD pathways. Our results showed that *Caspase-1*, a marker for canonical pyroptosis (Bergsbaken et al., 2009), was up-regulated in both α- and β-microglia cells (Figure 7A–D). In light of this, the expression of *NOD-like receptor (NLR) family, pyrin domain-containing protein 3* (NLRP3) and its downstream target *Gasdermin-D* (*GSDMD*) showed simultaneous activation in both forms of microglial subtypes (Figure 7B,D). In contrast, *Caspase-3* and *Caspase-7*, markers for apoptosis (McIlwain et al., 2015) were minimally activated in both α- and β-microglia cells (Figures 7B,D). Consistently, the TUNEL staining revealed sparse labeling of apoptotic cells in pineal sections at both 24 and 72 h post HIBD (Supplementary Figure S2).

Since the single cell sequencing analysis revealed preferential activation of pyroptosis associated factors in microglia cells (Figure 7A,C), we then assessed their changes at protein level in pineal glands from neonates receiving sham or HIBD surgeries. Consistent with sequencing results, the expression of pineal NLRP3, Caspase1 and GSDMD were significantly up-regulated at both 24 and 72 h post neonatal HIBD (Figure 7E,F). In addition, the Caspase-1-cleavage form of GSDMD, GSDMD-N, showed subsequent up-regulation (Figure 7E,F). In consistent with this, we discovered that the up-regulation of Caspase-1 preferentially co-localized with Iba1, a microglia specific marker on cryosections of the pineal gland at 24 and 72 h post neonatal HIBD (Supplementary Figure S3). Taken together, these results thus confirmed microglial pyroptosis as a component of pathophysiology of neonatal HIBD.

## Discussion

Both clinical and experimental data showed that the pineal gland undergoes pathological changes upon perinatal HIBD. To investigate the heterogeneity of injury induced cellular response, we performed 10x single cell sequencing of the developing pineal gland over the time course post HIBD. Within molecularly identified cell types, we first revealed a β to α subgroup conversion of pinealocytes, which were identified as mostly affected cell type upon injury. Next, we showed that distinct subtypes of astrocytes and microglia cells responded to the neonatal brain injury with differential expression of reactive markers. Compared to other cell types, we identified substantial reduction of both subtypes of microglia cells. In light of this, our results showed that canonical pyroptosis was involved in the programmed cell death of pineal microglia cells. To our knowledge, this study serves as a first example that delineates the molecular and cellular dynamics of the pineal gland post neonatal HIBD.

In rodent pineal gland, pinealocytes are divided into two subtypes, on the basis of their morphological differences, named as Type I and II or, alternatively, light and dark pinealocytes (Pevet, 1977; Calvo and Boya, 1984). Consistent with a previous pineal study of single cell sequencing (Mays et al., 2018), we identified two subtypes of pinealocytes marked by different expression levels of *Asmt*, the last step enzyme in melatonin synthesis. Molecularly, α pinealocytes that are equivalent to Type II pinealocytes express elevated level of *Asmt* and are more powerful to catalyze melatonin synthesis (Rath et al., 2016; Mays et al., 2018).

Our single cell sequencing results revealed that although the fraction of total pinealocytes showed no significant changes, the percentage of the subtype with higher *Asmt* expression was substantially increased at 24 and 72 h post HIBD (Figure 2). One possible reason is an β-to α-pinealocytes conversion, which will partially rescue the melatonin synthesis impacted by HIBD. The expression of Ki67 (Figure 2) or TUNEL staining (Supplementary Figure S2) showed no overt differences in both subtypes of pinealocytes between sham and HIBD groups. These results suggested that this conversion was achieved by neither cell proliferation nor apoptosis.

However, it remained unknown whether such molecularly identified β to α conversion is accompanied by morphological changes. Neither did us know if this change was temporary or permanent. In future studies, we will investigate these questions by performing *in situ* hybridization along with functional analysis.

In normal conditions, astrocytes are transcriptional and functional diverse to play specific roles in various CNS circuits (Ben Haim and Rowitch, 2017). Such heterogeneity is preserved in reactive astrocytes in different injuries and diseases (Escartin et al., 2021). Here, we identified continuous elevation of *Vimentin*, but not *GFAP*, in α- and β-astrocytes. Notably, although GFAP is a most widely used reactive marker, it is up-regulation is not a golden standard of astrocyte activation. For example, GFAP up-regulation is not observed in the cortex upon traumatic brain injury (Escartin et al., 2019). Unlike α- and β-astrocytes, γ-astrocytes are keeping dividing postnatally and showed up-regulation of MMP2, which has been reported to promote wound healing and motor functional recovery (Hsu et al., 2006). However, it remains unclear whether such up-regulation is age-dependent. A perhaps more intriguing question is whether and how molecular distinction between astrocyte subgroups correlates to their reactivity and roles in the pineal gland post neonatal HIBD.

Microglia activation has been identified in almost every injury or neurodegenerative disorders, both during neurodevelopmental and aged animals. However, whether and how distinct microglia populations are activated in an injury dependent manner is still unclear. In this study, we observed sustainable up-regulation of *Ccl4* and *Ccl3* in α-microglia cells (Figures 6B,D). In contrast to α-microglia cells, β-microglia cells showed a delayed up-regulation of *Ccl3*, but not *Ccl4* (Figures 6B,D). In light of this, *Ccl4* ^*+*^ population occupy a small fraction of developing microglia cells and represent both age-related and injury activated microglia expressing a number of inflammatory signals (Hammond et al., 2019). Notably, Ccl4 serves as the ligand of chemokine receptor type 5 (Ccr5) and is thus involved in regulating diverse populations of immune cells (Weber et al., 2001). It will be interesting to see whether and when pineal microglia activation will return to the homeostatic status, which is observed at 5 days post neonatal spinal cord injury (Li et al., 2020).

The reduction of microglia revealed by single cell sequencing could be caused by multiple reasons. First, our analysis showed that some canonical microglial markers such as *P2ry12* and *Cx3cr1* showed profound reduction at 72 h post HIBD (Figure 6). Although in consistent with previous findings (Salter and Stevens, 2017; Hammond et al., 2019), this could potentially affect the molecular identification of microglia when analyzing results from the single cell sequencing. In addition, the number of microglia in specific brain region is highly dynamic dependent on the extent and area of post injury inflammation. Our focus on PCD does not rule out other possibilities causing pineal microglia reduction upon neonatal HIBD.

Pyroptosis, unlike apoptosis, is a type of proinflammatory PCD (Bergsbaken et al., 2009).

An interesting phenomenon is that microglia/macrophages can also undergo pyroptosis upon hypoxic or traumatic brain injuries (McKenzie et al., 2018; Lee et al., 2019; Chang et al., 2020; Lv et al., 2020). When comparing to neurons, pyroptosis in microglia will further amplify the pathology of hypoxic or traumatic brain damages due to the release of neurotoxic and inflammatory factors such as cytokines and reactive oxygen species. For instance, in the case of multiple sclerosis, the release of TNF-α from activated myeloid cells contributes to GSDMD upregulation, which further promotes pyroptosis in oligodendrocytes and demyelination (McKenzie et al., 2018). Besides microglia, our data showed that other pineal cell types underwent pyroptosis post neonatal HIBD. It will be therefore interesting to evaluate roles of cell-type specific pyroptosis in future studies. Nevertheless, preventing pyroptosis *via* pharmacological treatments might alleviate pineal inflammation and play a beneficial role in its pathology upon neonatal HIBD.

In conclusion, our data provided comprehensive snapshots of how distinct cell types in the pineal gland respond to neonatal HIBD in a time dependent manner. These advances helped building a comprehensive view of the pineal pathophysiology upon neonatal injury and thus shed lights into the identification of novel targets to intervene circadian dysfunctions caused by perinatal hypoxic and ischemic conditions.

## Data Availability

The datasets presented in this study can be found in online repositories. The names of the repository/repositories and accession number(s) can be found below: https://www.ncbi.nlm.nih.gov/, PRJNA743566.

## Supplementary Material

Supplementary Figure S1 Histological analysis of neonatal HIBD. **(A,B)** Representative images of transverse brain sections and pineal glands (n=3 for both conditions) stained with TTC at 24 h **(A)** and 72 h **(B)** post HIBD, respectively. Scale bars for brain sections and pineal glands: 5 and 1 mm, respectively.

Supplementary Figure S2 Assessment of pineal cell apoptosis at different time points post HIBD. **(A-C)** Representative images of transverse sections of the pineal gland with TUNEL staining and quantification **(C)** at 24 h **(A)** or 72 h **(B)** post sham or neonatal HIBD, respectively. **(D)** Representative images of transverse sections of the pineal gland showing a negative control (PBS, left) and a positive control (DNase I treated, right) of the TUNEL staining. Scale bar: 200 μm. n=5,5 for sham and HIBD animals, 5 sections crossing the pineal gland were used for individual animals, Student’s t test, n.s., no statistical significance.

Supplementary Figure S3 Assessment of microglia pyroptosis at different time points post HIBD. **(A-B)** Representative images of transverse sections of the pineal gland with immunostaining against Iba1 and Caspase-1 at 24 h **(A)** or 72 h **(B)** post sham or neonatal HIBD, respectively. Arrowheads indicated co-labeling of Caspase-1 and Iba1. Scale bar: 200 μm. **(C)** Quantification of relative fluorescent intensity of Caspase 1 (left) and percentage of & Iba1^+^/ Caspase-1^+^ cells (right) in the pineal gland at 24 h or 72 h post sham or neonatal HIBD, respectively. n=3,3 for sham and HIBD animals, 5 sections crossing the pineal gland were used for individual animals, Student’s t test, **, P < 0.01.
