# Supplementary figures and images for: Profiling Temporal Changes of the Pineal Transcriptomes at Single Cell Level Upon Neonatal HIBD
Source: Front Cell Dev Biol. 2022 Mar 8;10:794012. doi: 10.3389/fcell.2022.794012 (PMC8958010; doi:10.3389/fcell.2022.794012)

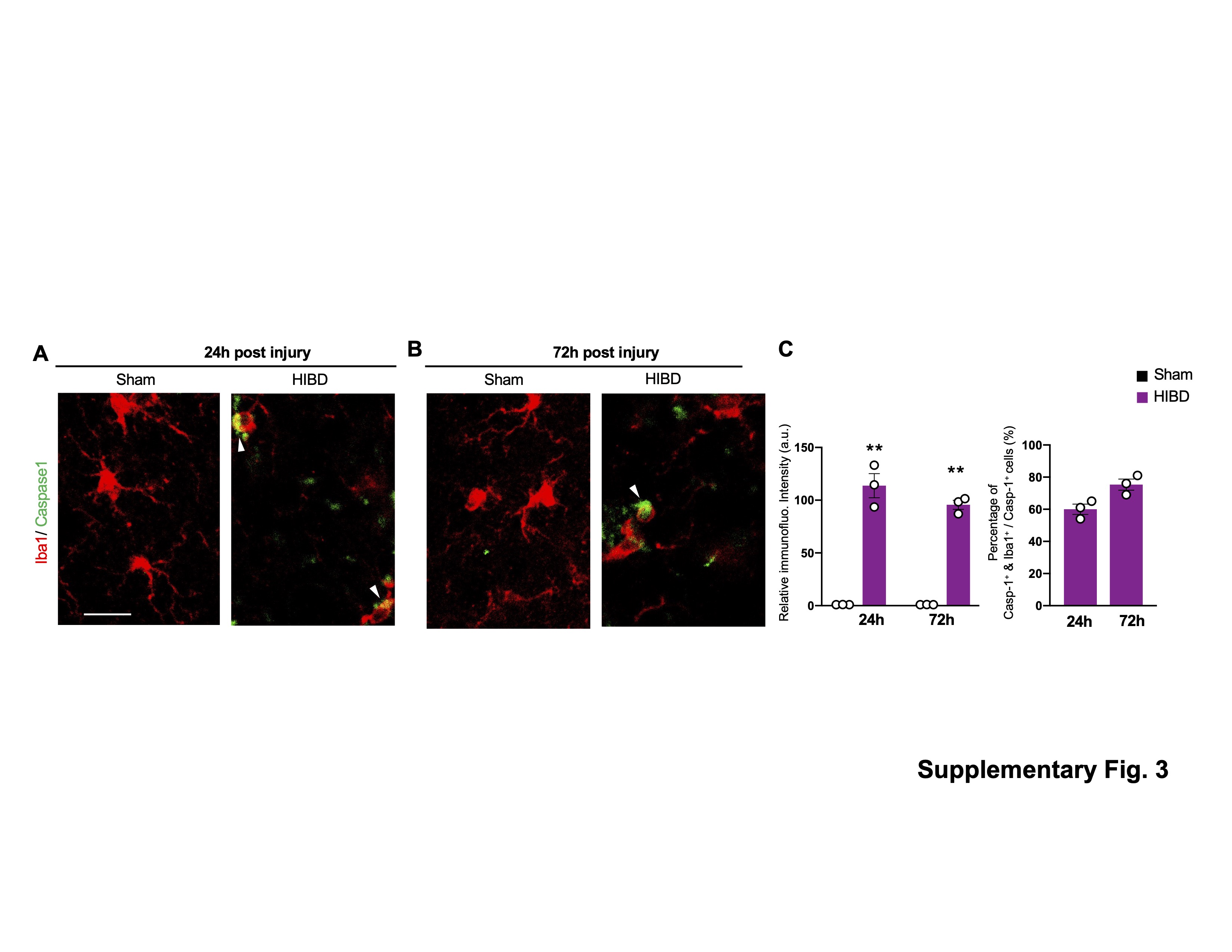

Supplement: Supplementary file 1 [file Image3.jpeg]

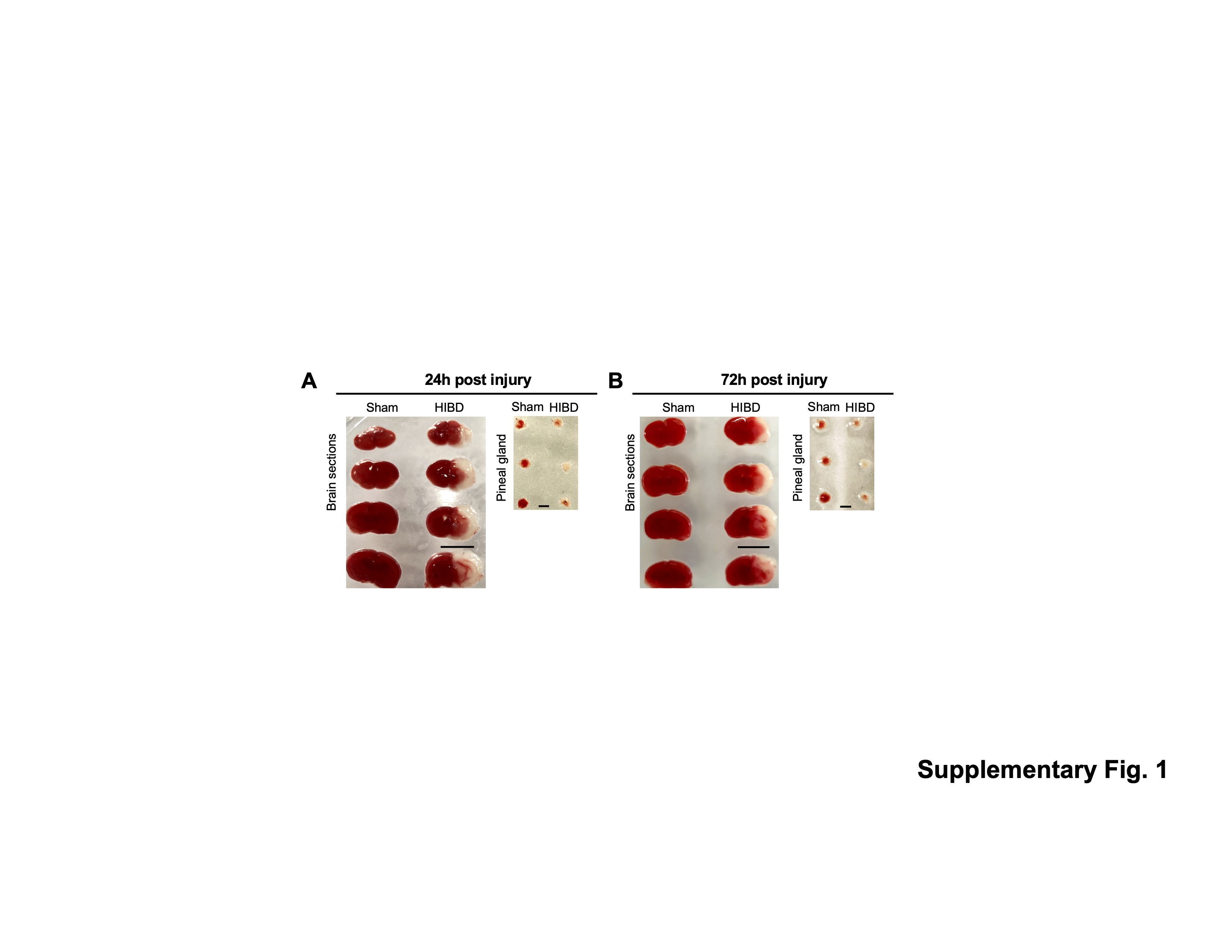

Supplement: Supplementary file 2 [file Image1.jpeg]

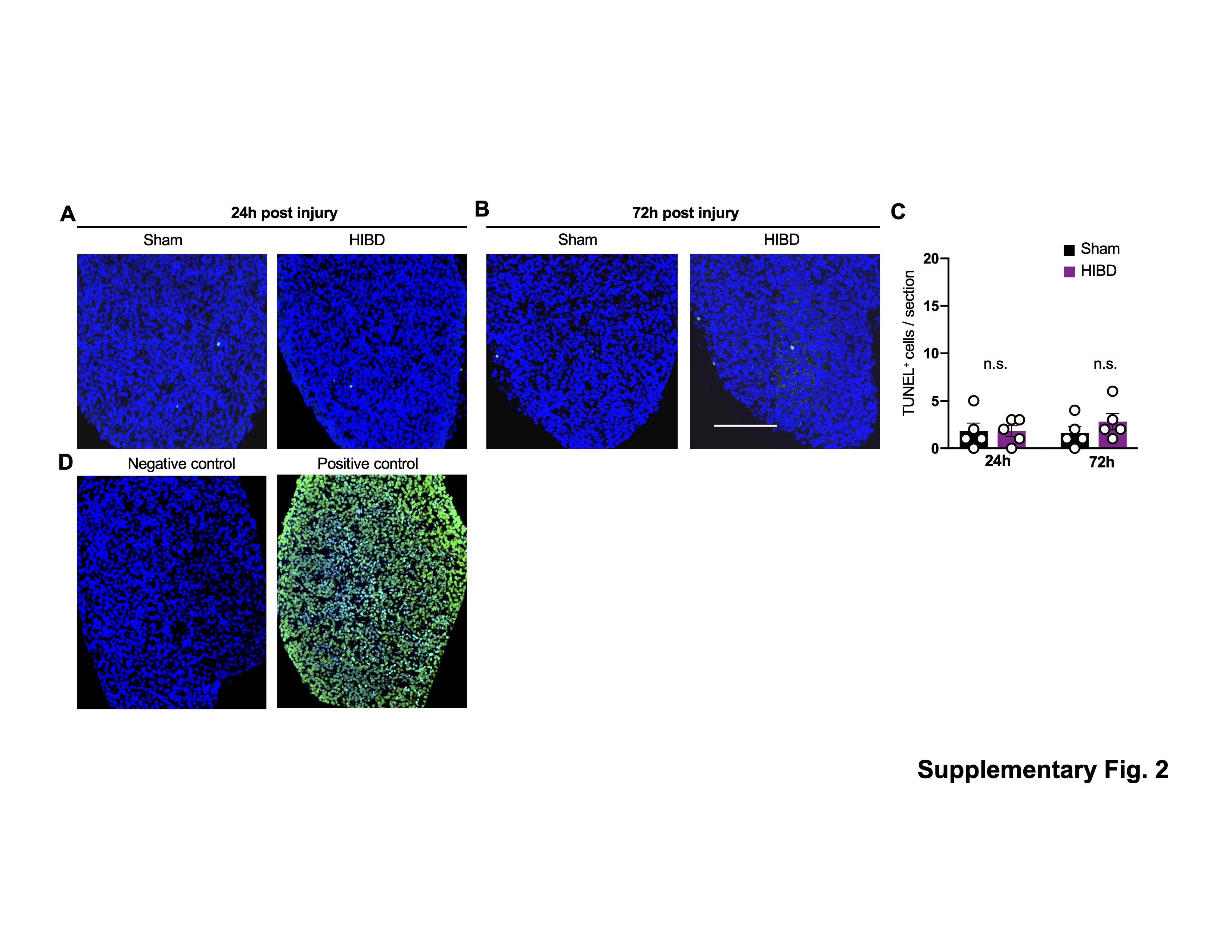

Supplement: Supplementary file 3 [file Image2.jpeg]
